# IWP-121: a novel sGC stimulator that reduces blood pressure and exhibits anti-fibrotic and anti-inflammatory activities in the Dahl Salt-Sensitive rat model

**DOI:** 10.1186/2050-6511-16-S1-A86

**Published:** 2015-09-02

**Authors:** Courtney Shea, Sheila Ranganath, Guang Liu, Derek Wachtel, Peter Germano, Jenny Tobin, Ping Zhang, Samuel Rivers, G-Yoon Jamie Im, James Sheppeck, Jaime Masferrer

**Affiliations:** 1 Ironwood Pharmaceuticals Inc, Cambridge, MA, USA

## Background

Soluble guanylate cyclase (sGC) is an intracellular receptor that can be activated by nitric oxide (NO) and sGC stimulators to produce cyclic guanosine monophosphate (cGMP), thereby modulating a number of downstream cellular and physiological responses including phosphorylation of VASP and vasodilation. In the Dahl Salt-Sensitive (DSS) rat model of hypertension, cGMP production by sGC is decreased, most likely due to reactive oxygen species (ROS) converting NO to peroxynitrite, resulting in depleted pools of NO available to bind to sGC. In this study we evaluated the efficacy of a novel sGC stimulator (IWP-121) in the DSS model. Male DSS rats (230-270 grams) received high-salt diet (8% NaCl) for 2 weeks followed by high salt plus compound for 6 additional weeks. IWP-121 was administered at doses of 1, 3, and 10 mg/kg/day in the chow (n=8/group). Losartan (30 mg/kg/day in the water) was used as a positive control, in addition to both High Salt (HS) and Normal Salt (NS). All groups were compared to HS control group for analyses.

IWP-121 dose dependently decreased mean blood pressure (MAP) throughout the study. Additionally, IWP-121 (at all doses tested) and losartan had statistically significant effects on decreasing heart hypertrophy and plasma NT-proBNP but only IWP-121 had an effect on attenuating liver hypertrophy. IWP-121 decreased microalbuminuria (an indicator of kidney end organ damage) as well as attenuated serum biomarkers known to be involved in inflammatory and fibrotic processes.

## Conclusion

In the rat DSS model of hypertension, there is a decrease in cGMP levels most likely due to the inactivation of endogenous NO by ROS. The sGC stimulator IWP-121, when administered in the diet exhibited sustained dose-dependent reduction in blood pressure. Additionally, IWP-121 attenuated heart and liver hypertrophy and reduced NT-proBNP, a biomarker of heart failure. The compound reduced levels of biomarkers for inflammation and fibrosis, and demonstrated renal end organ protection. sGC stimulators, like IWP-121 may have broad therapeutic application by modulating multiple relevant therapeutic endpoints including blood pressure, hypertrophy, inflammation, and fibrosis.

